# Wired in sync: Internet dependence in identical twins with detached personalities

**DOI:** 10.1192/j.eurpsy.2026.10444

**Published:** 2026-07-31

**Authors:** S. Khairwal, P. Gehlawat, B. B. Yadav

**Affiliations:** Psychiatry, Institute of human behaviour and allied sciences, Delhi, India

## Abstract

**Introduction:**

Internet Addiction Disorder (IAD) is an emerging behavioral addiction that causes significant impairment in academic, occupational, and social functioning (Young, 1998). It is classified under “Other Specified Disorders Due to Addictive Behaviours” in ICD-11 (WHO, 2019). Genetic and personality factors play an important role, with twin studies suggesting a heritable component (Li et al., 2016). Schizoid personality traits, marked by introversion, detachment, and preference for solitary activities, may further predispose individuals to excessive internet use (Dong et al., 2011). We present a case of monozygotic twins with IAD and concurrent schizoid personality traits, illustrating diagnostic and therapeutic challenges.

**Objectives:**

1. To describe the clinical presentation and management of internet addiction disorder in monozygotic twins.

2.To explore the role of schizoid personality traits as a comorbid vulnerability factor.

3.To discuss challenges in treatment adherence and therapeutic response in such complex cases.

**Methods:**

Two 20-year-old monozygotic twins were assessed at IHBAS. Diagnosis was based on ICD-11 criteria, Internet Addiction Test (IAT), and International Personality Disorder Examination (IPDE). Management included pharmacotherapy (fluoxetine, later bupropion), psychotherapy, occupational therapy, and phone-use restriction.

**Results:**

Both twins presented with:

•Excessive phone use (16–17 hours/day).

•Social withdrawal, poor self-care, and declining academic performance.

•Premorbid introversion and emotional detachment.

•Family history: mother with schizophrenia.

Clinical Findings:

•MSE: Shallow affect, preoccupation with phone retrieval, impaired judgment, poor insight (grade 1/5), intact perception.

•IAT: Confirmed severe internet addiction.

•IPDE: Presence of schizoid personality traits.

Management and Course:

•Initial treatment: Fluoxetine (20 mg) with behavioral therapy → poor compliance, no significant improvement.

•Revised treatment: Bupropion (150 mg OD → 150 mg BD), mobile phone restriction, occupational therapy (activity rescheduling), and psychotherapy.

•Response: Minimal improvement; patients showed persistent reluctance to change, reflecting underlying personality pathology.

**Conclusions:**

This case highlights the complex interplay between internet addiction disorder and schizoid personality traits. Treatment resistance, poor motivation, and non-compliance underscore the difficulty of managing behavioral addictions in individuals with personality vulnerabilities. Tailored multimodal approaches including pharmacological, psychological, and family-based strategies—are essential in similar cases.

**Disclosure of Interest:**

None Declared

